# An Evaluation of HTML5 and WebGL for Medical Imaging Applications

**DOI:** 10.1155/2018/1592821

**Published:** 2018-08-29

**Authors:** Qiusha Min, Zhifeng Wang, Neng Liu

**Affiliations:** Department of Digital Media Technology, Central China Normal University, Wuhan 430079, China

## Abstract

Despite the fact that a large number of web applications are used in the medical community, there are still certain technological challenges that need to be addressed, for example, browser plug-ins and efficient 3D visualization. These problems make it necessary for a specific browser plug-in to be preinstalled on the client side when launching applications. Otherwise, the applications fail to run due to the lack of the required software. This paper presents the latest techniques in hypertext markup language 5 (HTML5) and web graphics library (WebGL) for solving these problems and an evaluation of the suitability of the combination of HTML5 and WebGL for the development of web-based medical imaging applications. In this study, a comprehensive medical imaging application was developed using HTML5 and WebGL. This application connects to the medical image server, runs on a standard personal computer (PC), and is easily accessible via a standard web browser. The several functions required for radiological interpretation were implemented, for example, navigation, magnification, windowing, and fly-through. The HTML5-based medical imaging application was tested on major browsers and different operating systems over a local area network (LAN) and a wide area network (WAN). The experimental results revealed that this application successfully performed two-dimensional (2D) and three-dimensional (3D) functions on different PCs over the LAN and WAN. Moreover, it demonstrated an excellent performance for remote access users, especially over a short time period for 3D visualization and a real-time fly-through navigation. The results of the study demonstrate that HTML5 and WebGL combination is suitable for the development of medical imaging applications. Moreover, the advantages and limitations of these technologies are discussed in this paper.

## 1. Introduction

Internet technologies have evolved to a point where it is possible to create web-based applications that are comparable with those typically found on desktop computers and workstations. Web applications have been used in a wide range of areas, including business, media, education, and the medical community. Many radiologists recently participated in the development of these web applications for radiological purposes [1]. Examples of web-based medical imaging applications are presented briefly in Table 1.

Although these applications enable radiologists to share images and implement remote access interpretations, there are still certain technological challenges that need to be addressed. As can be seen from Table 1, Java is the most popular web technology for developing these applications owing to its cross-platform compatibility and remote accessibility [13]. Unfortunately, the implementation of Java is dependent on the preinstallation of the JVM. During the installation process, certain constraints such as administrative permission prohibit changes to the computer. In this case, Java-based web applications fail to run due to the unsuccessful installation of the JVM. This drawback leads to several disadvantages in web-based applications. Moreover, a similar problem exists with ActiveX applications. If this problem remains unsolved, radiologists may be unable to use the applications due to the lack of the required browser plug-in, thereby discouraging them from implementing remote access interpretations in the future.

Another challenge with respect to the development of medical imaging applications is the lack of an efficient approach for 3D visualization. Although recent studies have proposed the application of AJAX or TypeScript to develop web-based medical imaging applications to solve the plug-in problem, it can be seen from Table 1 that these types of applications lack three-dimensional (3D) visualization features. Given that a series of medical images is typically larger than 200 MB, the rendering of the entire dataset requires a significant amount of memory and time. Certain researchers have proposed the use of VRML to achieve hardware-accelerated rendering over the Internet; however, this presents three severe drawbacks, which are the plug-in problem, highly limited interface, and inconsistencies with different web browsers [20]. Due to the inherent limitation of the Internet technologies, web-based 3D visualization has recently become an impediment to the development of medical imaging applications.

In 2014, the World Wide Web Consortium (W3C) officially published a new version of HTML, referred to as HTML5 [21]. The previous version of HTML could only display simple information and implement simple interactions. These static web pages were not suitable for publishing variable data and were unable to provide rich interfaces over the Internet. To address this drawback, W3C updated the HTML standard. The latest version, HTML5, provides a richer and more interactive user experience with many new advanced features, including interaction with local files, image pixel operations, and support for advanced 3D functionalities. With the upgrades in the latest standard, HTML5 features are available in most browsers. A number of studies have therefore been conducted to exploit the potential of HTML5 in specific areas, such as e-learning [22] and remote macromolecular visualization [23]. In the field of medical imaging applications, Monteiro et al. used HTML5 to develop a sophisticated web-based medical image viewer [24]. The experimental results revealed that the application successfully demonstrated basic interpretation functions, for example, measurement, annotation, windowing, image rotation and filtering, and zooming. The performance of the HTML5-based medical image viewer was then improved by reducing the data access latency [25]. Although the HTML5-based implementation solved the plug-in problem, it lacked 3D features, which presents a potential limitation of the study. Many studies have confirmed the feasibility and clinical benefit of the 3D functions used in radiological interpretation [26]. In particular, with the assistance of the 3D function, the examiner performance with respect to sensitivity and interpretation time is better than that in the case wherein only a two-dimensional (2D) tool is used [27, 28]. Therefore, in addition to 2D image processing tools, the remote medical imaging application needs to provide 3D functions, to help the examiner make timely and accurate decisions.

Visualization researchers proposed WebGL [29] as a solution for web-based 3D visualization, due to its capability to access OpenGL for Embedded System 2 (OpenGL ES2) using a JavaScript application programming interface (API) [30–35]. The combination of the HTML5 <canvas> element and WebGL enables hardware acceleration, without plug-in requirements. WebGL was initially implemented at Mozilla in 2006 and the nonprofit technology consortium, Khronos Group, worked on the WebGL standard in 2009. In 2011, the WebGL1.0 specification was released, and most browsers supported it, for example, Chrome, Firefox, Safari, and Opera. Thus far, there are several WebGL demos that demonstrate its immense potential to incorporate 3D graphics into web pages [32, 33]. Hence, Cantor-Rivery and Peters described how these new technologies (HTML5 and WebGL) can improve medical imaging web applications, with respect to 3D visualization [36], and presented a demo that demonstrates an excellent performance of this combination [37]. Unfortunately, a comprehensive quantitative evaluation of the application running in different contexts was not provided. More tests are required to confirm the suitability of HTML5 and WebGL for the development of remote access medical imaging applications. It can therefore be concluded from the literature that HTML5 and WebGL are not being extensively explored by the medical community, and there is a need for a comprehensive qualitative and quantitative evaluation of these technologies.

In this paper, the latest revision of HTML (HTML5) combined with WebGL is discussed in the context of a potential solution to the plug-in and 3D visualization problems. As the new standard for the web, HTML5 is platform-independent, and in combination with the promising WebGL 3D web technology, it can create sophisticated 3D applications. The aim of this study was to evaluate the performance of an HTML5-based medical imaging application with respect to accessibility, functionality, and usability to determine its appropriateness for remote viewing and the interpretation of radiological images. The various functions of this application are covered in detail in the following sections, and a series of results are presented to confirm the suitability of HTML5 and WebGL for the development of remote access medical imaging applications.

## 2. Materials and Methods

### 2.1. Application Design

There are several common functionalities of medical imaging applications. First, it is preferable to initially transfer the medical dataset from the server to the client side so as to provide a short response time. Therefore, the application is required to directly interact with the local file system. Furthermore, to facilitate radiological interpretation, the basic functions for image processing are necessary for radiologists to identify the useful information contained in images. Finally, the interpretation may be supported by 3D functions so that the volumetric dataset can provide more details of patient anatomies and pathological conditions. Thus, a comprehensive medical imaging application should include the following features: (1) interaction with the local file system, (2) basic functions for 2D image processing, and (3) 3D visualization of the region of interest within the dataset.

**Figure 1:**
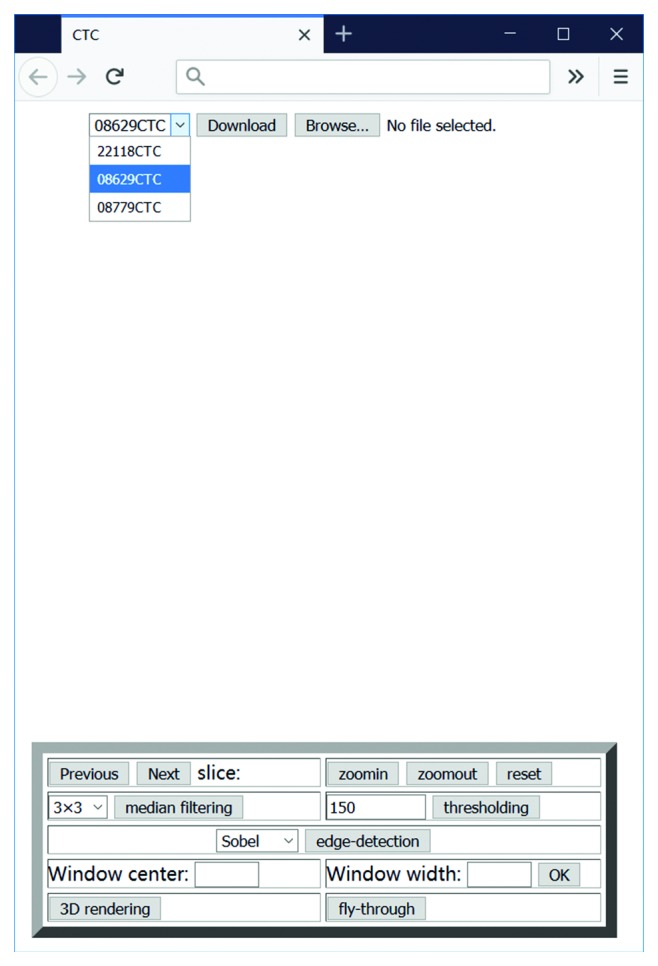
Screenshot of the initial start page for the demo application.

In this paper, a demo application for computed tomography colonography (CTC), also known as virtual colonoscopy, is designed to satisfy all criteria and is then used to conduct an evaluation of the HTML5 and WebGL combination as a development tool. The initial start page for the demo application is presented in Figure 1 and available at http://203.195.157.19/datatest/2D_Viewer.html. The operation flow of radiological interpretation using this demo application is as follows:

 
*Step 1*. Select a dataset to be interpreted and click the *Download* button, which activates the download process. The selected dataset is stored on the local computer.
 
*Step 2*. Click the *Choose file* button and in the dialog box, select the downloaded file. As a result, the first slice of the dataset is automatically displayed on the screen.
 
*Step 3*. Navigate through the image dataset using the *Previous* and *Next* buttons.
 
*Step 4*. Interpret the dataset using 2D image processing tools, for example, zoomin, zoomout, filtering, threshold, edge detection, and windowing.
 
*Step 5*. Interpret the dataset using 3D visualization tools, for example, 3D rendering and fly-through.

This application provides remote access interpretation in such a way that radiologists can view images from a downloaded dataset and manipulate them using 2D or 3D functions.

This application can be placed as a client component in a large teleradiology system. A typical teleradiology system comprises two components: the server side and the client side of the application. The server-side component is responsible for retrieving the required image datasets and sending them to the client. The client side is a medical imaging application. The application sends the request to the server to download the selected dataset and displays the downloaded image. The radiological interpretations can be assisted by the 2D and 3D functions in the application.

This paper focuses solely on the client application and presents an evaluation of HTML5 and WebGL for the development of medical imaging applications. The following section presents a demo application for CTC and a discussion on all the necessary implementation details.

### 2.2. Application Implementation

#### 2.2.1. Access to the Local File System

The application enables the user to choose a patient dataset to study (Figure 1). The selected dataset is transmitted to the client side and stored on the local computer using a custom format. At present, HTML5 has added a new input type, <input type = “file”>, which provides a standard way to interact with local files. The File Open dialog box that appears when the user clicks the *File* button is used to access the local file system from the browser. This new feature in HTML5 is a core requirement for the remote viewer application.

**Figure 2:**
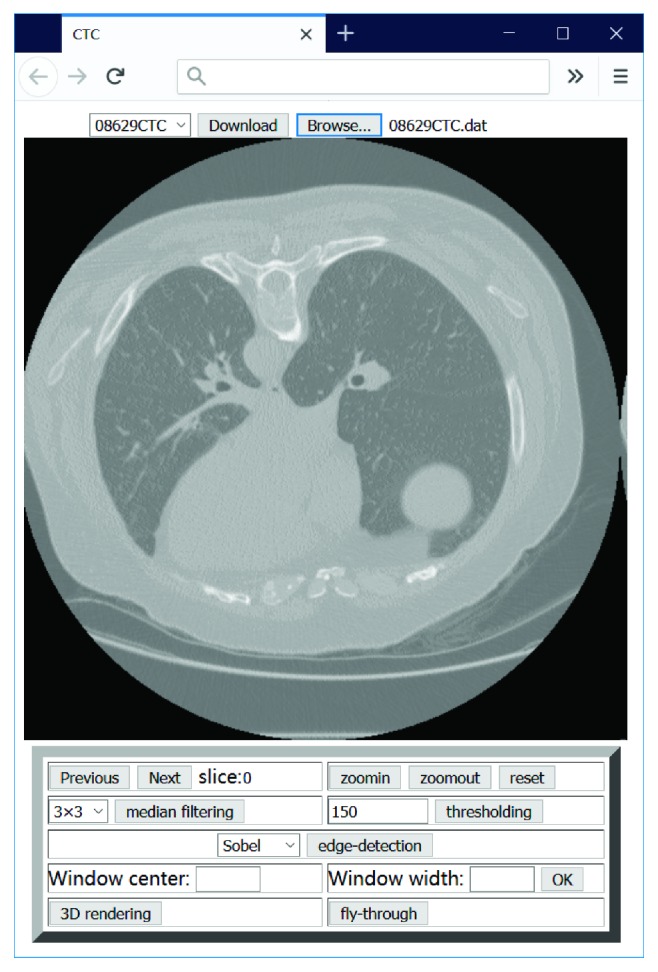
Screenshot of an image display.

Once the downloaded file has been opened, the first slice in the dataset is automatically displayed on the screen (Figure 2).

#### 2.2.2. Image Processing

**Figure 3:**
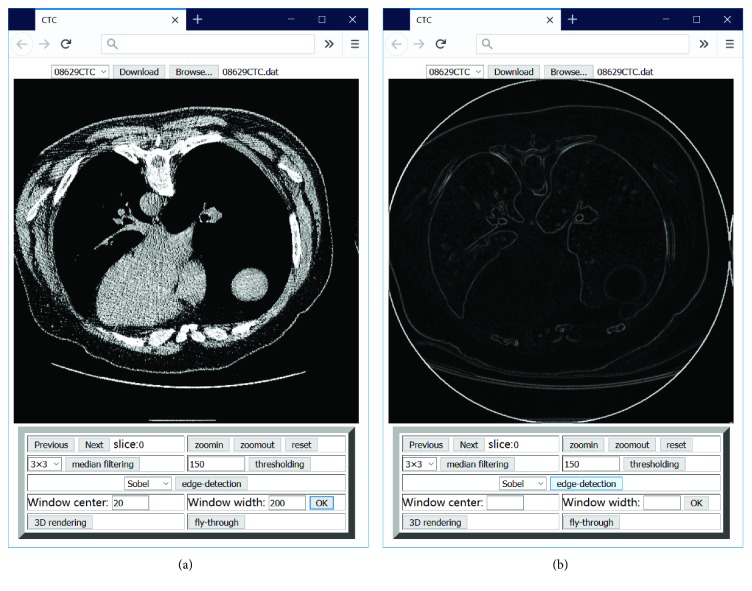
Screenshots of the user interface of the implementation of typical image processing functions: (a) a slice with adjusted window (parameters: center = 20 HU, width = 200 HU); (b) edge detection using the Sobel operator.

Another crucial requirement for this application is pixel-level manipulation. Owing to the introduction of the <canvas> element in HTML5, it is possible to define the color of an image pixel in the canvas. The application is therefore capable of implementing certain advanced image processing functions that could not be achieved with previous versions of HTML. 2D image processing functions, for example, magnification, windowing, filtering, thresholding, and edge detection are provided by the application and typical functions depicted in Figure 3. The implementation of accessing the local file and 2D image processing comprises approximately 700 lines of JavaScript code without support from other libraries.

#### 2.2.3. 3D Visualization

It is well known that 3D visualization is extremely computationally intensive. Hence, this task is normally implemented at a workstation equipped with a high-performance graphics processing unit (GPU). For a single CTC interpretation, the application is required to render approximately 1,000,000 polygons to generate the entire colon surface. As is expected, it is difficult to complete this 3D visualization on a personal computer (PC) that is not equipped with high-performance GPUs or to run it in a browser.

Based on the study by Cantor-Rivery and Peters [36, 37], in this study, WebGL was used for 3D functions realized using HTML5 implementation. In addition, WebGL is the most promising technology to satisfy the criteria, which are as follows:

- Accessibility: the application should be supported by most web browsers, and the interface should require no more than the interaction capabilities of a standard PC. In this way, users can complete the remote access interpretation using their existing computers, without any additional software or hardware requirements.
- Functionality: the application should provide a 3D visualization for the region of interest within the dataset.
- Usability: the application should be easy to use, and a short response time is an important factor that affects the user satisfaction.

The following presents the 3D implementation based on HTML5 and WebGL used in this study.

**Figure 4:**
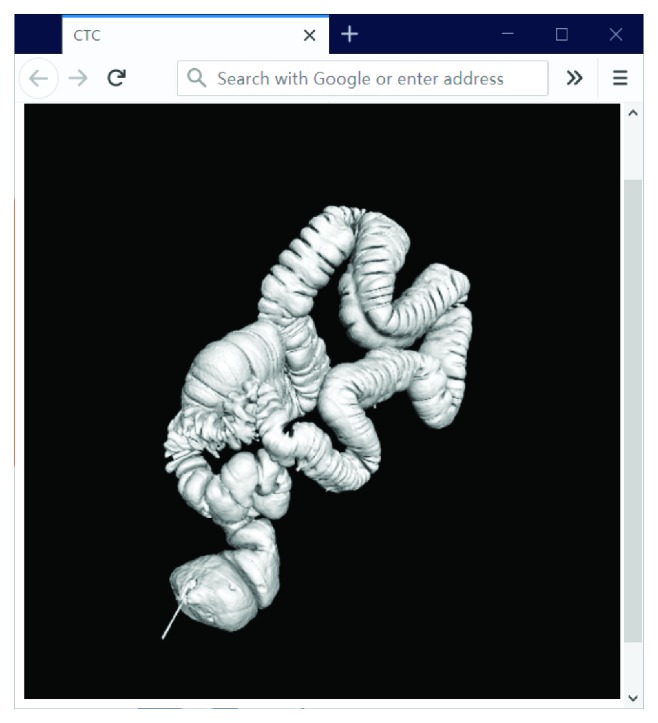
Screenshot of a 3D model of the whole colon in the browser.

In this implementation, the 3D visualization is based on surface rendering. Surface rendering generally involves two stages: surface extraction and 3D rendering. The marching cubes algorithm [38] is used to extract the isosurface from a volumetric dataset. The preferred method for shortening the rendering time is to perform the surface extraction once and store the vertex and normal files on the server. When the user sends the request to view the 3D data, the application loads the corresponding vertex and normal files and renders the 3D content on the client side, without the need to perform surface extraction. Once the vertex and normal files of a 3D model have been set, the 3D model surface can be defined and WebGL renders the entire model according to the predefined light and viewpoint. The total number of lines of JavaScript code for 3D rendering is approximately 500, without support from other libraries.  Figure 4 presents a 3D colon model extracted from a CTC dataset using the application described above. The user can also interact with this model and perform operations such as rotation and translation using the mouse.

**Figure 5:**
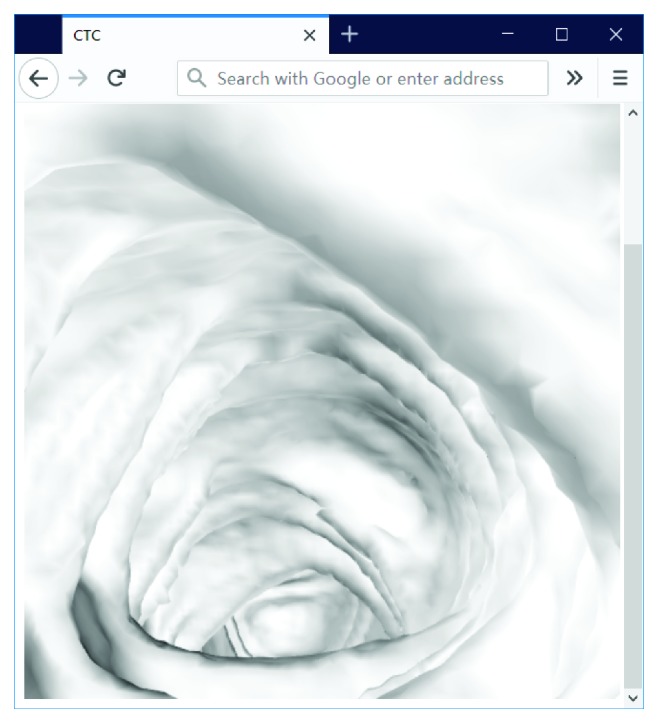
Screenshot of the implementation of the fly-through in the browser.

Virtual fly-through navigation is a feature used in manipulating the results of the 3D reconstruction. Moreover, WebGL provides a function to set the viewpoint location, which is an essential prerequirement for the camera movement. The camera can therefore move along a planned path, commonly referred to as the colon center line, to render internal views. Using these advanced imaging techniques, the radiologist can examine the inner wall using 3D fly-through in a virtual colon model which has been regarded as a time-efficient method for colon cancer detection. The same CTC dataset was used to implement a fly-through function.  Figure 5 presents a captured image of the 3D fly-through within the colon, running in a browser.

## 3. Results

### 3.1. Experiment Design

The demo application enabled radiologists to connect to an image server to download the selected dataset and then manipulate the images in a web browser. A range of appropriate tools, such as windowing, zooming, and 3D visualization, were also provided by the application.

To evaluate the HTML5-based application, two types of experiments were conducted. The first was used to determine the performance of the application on multiple platforms. The second was used to evaluate the performance of the application using either a local area network (LAN) or a wide area network (WAN). Three datasets were used in the experiments. The data were downloaded from the Cancer Imaging Archive (TCIA), which provides a freely accessible and open archive of cancer-specific medical images to the research community [39]. A complete description of the datasets is presented in Table 2, and the information about computers used in the experiments is presented in Table 3. It is evident that the computers were ordinary laptops for regular users.

Thus far, all major browsers including Firefox, Chrome, Safari, IE, and Microsoft Edge support HTML5 and WebGL. However, when testing the application in different browsers, IE returned a memory error, due to the large number of faces that required rendering. Therefore, the experiments were focused on Chrome, Firefox, Safari, and Microsoft Edge. The details of the testing metrics in this study are presented in Table 4.

### 3.2. Performance on Multiple Platforms

**Figure 6:**
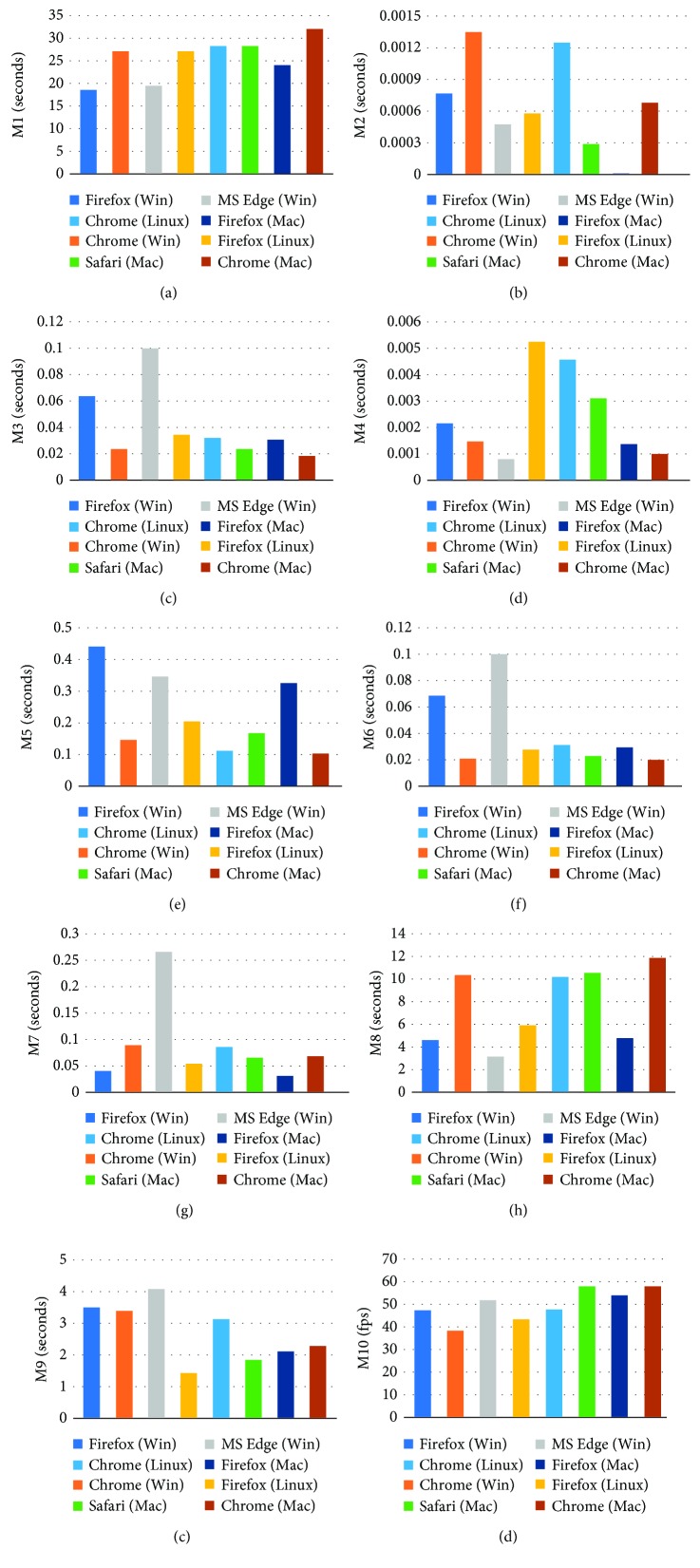
Application performances on different platforms. The computational time required for (a) downloading the selected dataset; (b–g) implementing 2D image processing per slice; (h–j) implementing 3D visualization.

The first experiment was carried out using dataset 1 through LAN to evaluate the performance of the application on multiple platforms. The demo application was run on several computers to test the performance on the Windows, Linux, and Mac platforms. Each function in the application was implemented 20 times on these platforms, using different browsers. The average performances for each function are presented in Figure 6. A comparison of the performances on the different platforms reveals that Windows, Linux, and Mac could provide nearly the same application performances, with the exception of magnification and 3D rendering. Magnification implemented on Linux was much slower than that implemented on Windows and Mac, regardless of the browsers used; whereas 3D rendering implemented on Linux and Mac was much faster than that implemented on Windows. Although different platforms could provide nearly the same application performances, the performance of each browser on different platforms led to inconsistencies. For example, Firefox on Windows and Mac provided faster windowing than that on Linux, whereas Firefox on Linux and Mac provided faster thresholding than that on Windows. Additionally, the inconsistent performances on different platforms also occurred in Chrome. For example, the execution time for viewing a slice on Windows and Linux was twice that on Mac and the execution time for magnification on Linux was twice that on Windows and Mac. However, on Mac, Safari, Firefox, and Chrome provided nearly the same 2D and 3D performances.

### 3.3. Performance via LAN and WAN

In the second experiment, the computer was used to test the application over LAN and WAN. This computer was equipped with Windows, and therefore had only three browsers, that is, Microsoft Edge, Firefox, and Chrome, which were used to implement the HTML5-based application. All three datasets were used in the experiment. Dataset 1 was used to identify which of the three browsers could offer the best performance for the HTML5-based application. Dataset 2 was used to determine the performance differences in Chrome when running the application via LAN and WAN. Dataset 3 was used to determine the performance differences in Firefox when running the application via LAN and WAN. In WAN, the application accessed the medical image dataset and vertex/normal files on the remote server. The bandwidth of the connecting network was 40 Mbps, and it had a download speed of approximately 4.8 MB/s. The download sizes for the medical image dataset, vertex file, and normal file are presented in Table 2.

Each function in the application was implemented 20 times by Chrome, Firefox, and Microsoft Edge, either over the LAN or WAN. The average performances for each function are presented in Table 5. The results of the performance tests of Chrome, Firefox, and Microsoft Edge on dataset 1 revealed that Firefox and Chrome were superior to Microsoft Edge with respect to 2D image processing. However, for 3D visualization, Microsoft Edge achieved the highest frame rate with an increase of 20% when compared with Firefox and 50% when compared with Chrome. Furthermore, the results presented in Table 5 reveal that the most significant difference between the LAN and WAN was the data transmission speed, as expected. After loading data to the local storage, the application performance over the WAN was the same as that over the LAN.

The experiments revealed that the response time for the 2D image processing functions was significantly less than 1 s per slice on every computer. It can therefore be concluded that the application can demonstrate real-time performances for all the provided 2D tools. For the 3D visualization, approximately 1 min was required for the download of the vertex and normal files and the generation of the entire 3D model over the WAN, whereas over the LAN, approximately 14 s (execution time on M8 and M9) was required. The user was then able to control the camera movement, resulting in the change of viewpoint in real time.

## 4. Discussion

At present, three alternatives to HTML5 for the development of web applications are Flash, Java, and Silverlight [40]. Circa 2000, Java was a popular web technology used in teleradiology applications. Slomka et al. developed a remote viewer using Java applets [2]. In their system, the compressed patient images were downloaded with a Java archive file (JAR); thus, the client required only one connection to the server, which can dramatically reduce network traffic, due to the decrease in the client-server communications. Knoll et al. [3] and Choi et al. [4] also developed Java applications for teleradiology purposes. Recently, the ubiquity and small size of Flash Player stimulated a growing trend toward the utilization of Flash. Arguiñarena et al. used this type of application to connect with a picture archiving and communication system and to provide a shorter image display time to improve the teleradiological productivity [41]. Our previous study determined the suitability of Flash for the development of web-based medical imaging applications [42].

However, the main disadvantage associated with these technologies is the requirement of a browser plug-in. Although Flash Player is the most popular browser plug-in technology in the world thus far; in 2010, Apple decided to stop incorporating Flash Player in Macs. For this group of users, it is therefore necessary to ensure that their computers are equipped for running Flash applications, which requires an initial installation of the Flash Player. Moreover, the same problem occurs in Java and Silverlight. To run Java-based web applications without a plug-in installation, Oracle developed a tool to generate the package for a self-contained application. This application contains the Java application and the required Java Runtime Environment and requires no additional JVM installation. However, the drawbacks of self-contained application packages are their lager download sizes and platform dependencies [43]. Fortunately, these drawbacks do not exist with the application described in this paper, given that HTML is the native language of all browsers. Therefore, HTML5 is a truly “no preinstallation” and platform-independent technology.

Another drawback of remote access medical applications is the visualization of the region of interest within a volumetric dataset. The majority of the previous work was focused on the utilization of VRML on the client side [7, 10]. However, this approach also suffers from several limitations, which were discussed in the previous section. This study addressed a different technology for an improvement in user friendliness. Furthermore, the WebGL and HTML5 combination has numerous potential advantages over VRML with respect to JavaScript support, consistency across different browsers, and no plug-in requirements. Cantor-Rivery and Peters demonstrated that the HTML5 and WebGL combination for medical imaging applications could solve the problem relating to the inability of user browsers to support 3D interactions [37]. The demo application was tested on several platforms and required only 1 min to download the corresponding files and generate an entire 3D model while running on an ordinary laptop computer in a remote location. In addition, as a useful tool for the image interpretation, virtual fly-through navigation could also be implemented in real time using a browser. It should be noted that the download sizes of the vertex and normal files could be dramatically reduced by server-side smoothing and polygon decimation, thus shortening the download time for the vertex and normal files. On the other hand, if the clients are high-performance computers, the 3D reconstruction process can be implemented locally, which eliminates the necessity of downloading the vertex and normal files. Furthermore, all these procedures make it possible to further improve the 3D performance of the application.

Nevertheless, there are minor limitations in the current implementation. At the time of writing, the latest version of HTML released was 5.2. In comparison with HTML5.0, the latest version provides new features, such as the <dialog> element and the allowpaymentrequest attribute of the <iframe> element. However, the method for accessing the local file in HTML5.2 still requires user interaction. Therefore, in the application in this study, the users themselves are required to specify the file path when reading the downloaded dataset. Given that the W3C is still working on the HTML5 specification, in the future, a smart way may be developed in HTML5 to read the local file after obtaining user permission, such as trusted applications in Java and Silverlight. Furthermore, the 3D visualization of the medical image data is based on surface rendering, and the surface information is predefined on the server. Consequently, the user is not allowed to choose the surface-generation parameters, and the parameters may affect the geometric representation of the 3D models. To provide a custom 3D visualization of medical image data, it is reasonable to allow the user to define the surface-generation parameters in the application, and the server then implements a fast surface extraction according to the chosen parameters. In this case, a further study on the fast surface extraction is required. One more limitation of this study is browser compatibility. Given that the W3C HTML5 recommendation was released in 2014, old versions of browsers do not support HTML5. Therefore, some of the latest features in HTML5 may not be compatible with user browsers (e.g., the <canvas> and <video> elements), if the browsers are not updated. However, as HTML5 is used more extensively, it is expected that all of the browsers installed on PCs will support HTML5, and the medical community will adopt the more advanced features of HTML5. The results of the comparison with other teleradiology applications suggest that this new type of web application can provide the necessary functionalities for radiological interpretations and offer an excellent performance for remote access users, especially in a short time, for 3D visualization and real-time fly-through navigation.

## 5. Conclusions

From a review of the literature, it is apparent that there is a need for a straightforward solution to the remote access of radiological images. As revealed in this paper, an HTML5-based application provides a solution by the remote implementation of 2D image processing functions and 3D visualization without the need for preinstallations. Moreover, it should be noted that the implementation of the application only requires a web browser on an ordinary computer. The experimental results indicate that this solution can achieve real-time performances for 2D and 3D functions over both LAN and WAN, providing significant improvements with respect to accessibility, functionality, and usability. Therefore, it can be concluded from the study that the HTML5 and WebGL combination can provide a remote access medical imaging experience that is comparable with (or superior to) that of alternative technologies that are currently available.

## Figures and Tables

**Table 1:** An overview of several previously published medical imaging applications.

Group	Year	Client technology	Functions	Required plug-in
Slomka et al. [2]	2000	Java	Access to remote patient database, the compressed image is transmitted to the local computer, and several applets are available for different study types	JVM^a^
Knoll et al. [3]	2000	Java	Patient administration, 2D reconstruction and image processing, interpretation and collaborative diagnosis	JVM
Choi et al. [4]	2002	Java	Medical imaging conference	JVM
Zeng et al. [5]	2003	ActiveX	Access to remote medical image databases, image presentation, and image processing	ActiveX plug-in
Kaldoudi and Karaiskakis [6]	2006	XML	Access to remote medical image databases and image presentation	None
Mongeau et al. [7]	2008	VRML^b^	3D visualization	VRML browser plug-in
Kamauu et al. [8]	2008	Java	Image display and processing	JVM
Costa et al. [9]	2009	ActiveX	Access to remote medical image databases and image presentation	ActiveX plug-in
Mahmoudi et al. [10]	2010	AJAX^c^ + VRML	Image processing; and 3D visualization	VRML browser plug-in
Shen et al. [11]	2014	AJAX	Access to remote medical image databases and image presentation	None
Qiao et al. [12]	2015	AJAX	Image presentation and image processing	None
Looney et al. [13]	2016	Java	Access to remote medical image databases and image processing	JVM
Xiberta and Boada [14]	2016	AJAX	Image presentation and image processing	None
Doel et al. [15]	2017	Java	Medical imaging data sharing	JVM
Jiang et al. [16]	2017	Android	Transmission performance improvement with respect to batch transmission processing and image presentation	None
Huang et al. [17]	2018	AJAX	Access to remote medical image databases and image presentation	None
Gøeg et al. [18]	2018	TypeScript [19]	Access to remote medical image databases and image presentation	None

^a^JVM is the acronym for Java virtual machine; ^b^VRML is the acronym for virtual reality modeling language; ^c^AJAX is the acronym for asynchronous JavaScript and XML.

**Table 2:** Description of the datasets used in the experiments.

Dataset	Type	Size	3D visualization		
			Vertex file	Normal file	The number of faces
#1	CT	512 × 512 × 610 (312,320 kB)	59,545 kB	59,545 kB	1,693,700
#2	CT	512 × 512 × 628 (321,536 kB)	57,747 kB	57,747 kB	1,642,580
#3	CT	512 × 512 × 500 (256,000 kB)	60,236 kB	60,236 kB	1,713,372

**Table 3:** The details of computers using in the experiments.

Computer	Type	Operation system	CPU	Memory	GPU
I	Laptop	Windows 10 64-bit	Intel(R) Core(TM) i5-6300HQ CPU @ 2.30 GHz	8.00 GB	NVIDIA GeForce GTX 950M
II	Laptop	Ubuntu 16.04	Intel(R) Core(TM) i5-6300HQ CPU @ 2.30 GHz	8.00 GB	NVIDIA GeForce GTX 950M
III	Laptop (MacBook Pro)	Mac OS Sierra 10.12.5	Intel Core Intel i5 @ 2.30 GHz	8.00 GB	Intel Iris Plus Graphics 640

**Table 4:** The details of testing metrics in this study.

Function	Label	Description	Measurement
Data access	M1	Execution time for downloading a medical image dataset	Manual measurement
2D image processing	M2	Execution time for viewing a slice in a medical image dataset	Measured by JavaScript code
	M3	Execution time for implementing windowing per slice	Measured by JavaScript code
	M4	Execution time for implementing magnification per slice	Measured by JavaScript code
	M5	Execution time for implementing median filtering (3 × 3) per slice	Measured by JavaScript code
	M6	Execution time for implementing thresholding per slice	Measured by JavaScript code
	M7	Execution time for implementing Sobel edge detection per slice	Measured by JavaScript code

3D visualization	M8	Execution time for downloading the vertex and normal files of a medical image dataset	Manual measurement
	M9	Execution time for rendering a 3D model based on the downloaded vertex and normal files	Measured by JavaScript code
	M10	Frame rate of fly-through	Measured by JavaScript code

**Table 5:** Comparison of the proposed application performances over the WAN and LAN.

Dataset	Browser	Data access	2D image processing						3D visualization		
		M1 (s)	M2 (s)	M3 (s)	M4 (s)	M5 (s)	M6 (s)	M7 (s)	M8 (s)	M9 (s)	M10 (fps)
#1	Firefox (WAN)	18.6	0.0008	0.066	0.002	0.456	0.070	0.040	4.7	3.61	49.54
	Chrome (WAN)	27.1	0.0014	0.024	0.002	0.150	0.021	0.089	10.5	3.52	39.8
	Microsoft Edge (WAN)	19.5	0.0005	0.102	0.001	0.359	0.102	0.268	3.2	4.12	60.0

#2	Chrome (LAN)	28.9	0.0013	0.025	0.001	0.167	0.024	0.103	10.4	3.30	40.41
	Chrome (WAN)	135.5	0.0015	0.029	0.001	0.169	0.028	0.165	46.0	3.78	40.05

#3	Firefox (LAN)	17.9	0.0003	0.039	0.001	0.468	0.039	0.039	4.8	3.80	47.28
	Firefox (WAN)	102.3	0.0005	0.042	0.002	0.495	0.046	0.068	40.2	3.85	46.95

## Data Availability

Three datasets used in the experiments were downloaded from The Cancer Imaging Archive (TCIA), which provides a freely accessible and open archive of cancer specific medical images to the research community.
